# Association of Serum Bilirubin and Lipid Ratio (Total Cholesterol/(High-Density Lipoprotein + Bilirubin)) in Coronary Artery Disease: A Case-Control Study at a Tertiary Care Hospital

**DOI:** 10.7759/cureus.46420

**Published:** 2023-10-03

**Authors:** Chiradeep Adepu, Bandakadi Sandeep Kumar Reddy

**Affiliations:** 1 Department of General Medicine, Osmania Medical College and Osmania General Hospital, Hyderabad, IND; 2 Department of Medicine, Osmania Medical College and Osmania General Hospital, Hyderabad, IND

**Keywords:** high-density lipoprotein, low-density lipoprotein, atherosclerosis, lipid profile, cardiovascular risk, lipid ratios, coronary artery disease

## Abstract

Introduction: Coronary artery disease (CAD) is a leading cause of morbidity and mortality worldwide, particularly in industrialized societies. The aim of the study was to investigate the potential association between lipid ratios and CAD risk and explore their diagnostic performance compared to traditional lipid profile parameters and total bilirubin levels.

Methods: A total of 50 cases with CAD and 50 controls without CAD were recruited. Clinical data, including age, gender, comorbidities, blood pressure, glucose levels, smoking history, cardiovascular examination findings, and electrocardiogram (ECG) results, were collected. Lipid profile parameters (total cholesterol (TC), high-density lipoproteins (HDL), low-density lipoproteins (LDL), and triglycerides) and total bilirubin levels were measured. Lipid ratios, including cholesterol (CHO)/HDL, LDL/HDL, HDL + bilirubin, LDL/(HDL + bilirubin), and TC/(HDL + bilirubin), were calculated.

Results: Significant differences were observed between cases and controls for comorbidities, including hypertension, diabetes, and obesity (p = 0.025), and ECG findings (p < 0.001). Lipid profile parameters were significantly different between cases and controls (p < 0.001). Lipid ratios also showed significant differences (p < 0.001) and demonstrated high sensitivity and specificity in identifying CAD. Among the ratios, LDL/HDL had the highest area under the curve (AUC) of 0.977, followed by CHO/HDL (AUC = 0.913), LDL/(HDL + bilirubin) (AUC = 0.903), and TC/(HDL + bilirubin) (AUC = 0.807). Total bilirubin alone did not show a significant association with CAD (AUC = 0.590, p = 0.119).

Conclusion: Lipid ratios (CHO/HDL, LDL/HDL, HDL + bilirubin, LDL/(HDL + bilirubin), and TC/(HDL + bilirubin)) showed promising potential as predictors of CAD, outperforming traditional lipid profile parameters and total bilirubin levels. These ratios could serve as valuable diagnostic tools in identifying individuals at higher risk of CAD.

## Introduction

Coronary artery disease (CAD) is a leading cause of morbidity and mortality worldwide, particularly in industrialized societies. Atherosclerotic plaque formation in the epicardial coronary arteries due to risk factors such as high low-density lipoprotein (LDL), low high-density lipoprotein (HDL), cigarette smoking, hypertension, and diabetes mellitus plays a crucial role in the pathophysiology of CAD [1-3]. Lipid oxidation and the generation of oxygen free radicals are implicated in plaque formation and atherosclerosis, making antioxidants potentially protective factors against CAD [4,5].

Bilirubin, a lipophilic antioxidant, has been found to be more effective in protecting lipids from oxidation compared to water-soluble antioxidants like glutathione [6,7]. Higher bilirubin concentrations within the reference range have been associated with protection against CAD, while lower concentrations indicate an increased risk of atherogenesis [8,9]. Additionally, serum bilirubin positively correlates with the protective factor, i.e., high-density lipoprotein cholesterol (HDL-C), enhancing the predictive value of major lipid risk factors. Ratios such as low-density lipoprotein cholesterol (LDL-C)/(HDL-C + bilirubin) and total cholesterol (TC)/(HDL-C + bilirubin) have shown promise in identifying severe CAD more accurately than traditional lipid ratios [10].

While there is evidence suggesting an inverse correlation between plasma bilirubin concentration and CAD morbidity, further investigation is warranted to establish bilirubin as a potential diagnostic marker for atherogenic risk.

The primary aim of this study is to compare the serum bilirubin levels and the ratio of TC/(HDL + bilirubin) in patients with and without CAD in a tertiary care hospital.

## Materials and methods

Study design

This is a case-control study conducted at Osmania General Hospital, Hyderabad, Telangana, India, between December 2020 and December 2022, with ethical approval obtained from Osmania General Hospital (OSM/IEC/2020/231).

Study population and sample size

The choice of a 1:1 case-control ratio with 50 cases and 50 controls was determined based on practical considerations and the availability of study participants. The study included 100 patients divided into two groups. Cases consisted of 50 patients with angiographically confirmed CAD, and controls consisted of 50 patients who underwent angiograms and were found to be CAD-free.

As part of the inclusion criteria, patients with angiographically confirmed CAD diagnoses were included as cases. Exclusion criteria include the following: (1) patients with serum bilirubin levels outside the normal range: this exclusion criterion likely aimed to focus on individuals with bilirubin levels within the typical range to investigate its association with CAD; (2) patients with known liver diseases or pathologies: this criterion helps ensure that liver-related conditions do not confound the study's findings regarding serum bilirubin levels and CAD; (3) individuals with left ventricular failure and a specific ejection fraction below 35%: this exclusion criterion likely aimed to isolate the impact of CAD on the study outcomes, as severe left ventricular failure can have independent effects on cardiovascular health; (4) patients with hematological conditions that are known to affect serum bilirubin levels were excluded: this criterion helps ensure that any observed associations between bilirubin levels and CAD are not influenced by underlying hematological disorders.

Data collection

After obtaining ethical committee clearance and informed consent, demographic data were obtained from all the subjects, including age, gender, comorbidities, blood pressure, glucose levels, and smoking history. A general and systemic examination was conducted on all study subjects, including laboratory investigations. A thorough cardiovascular system examination was performed using a pre-formed study proforma. The following tests were carried out on all patients, including a complete blood profile, liver function tests, a fasting lipid profile, an electrocardiography (ECG), two-dimensional echocardiography, and coronary angiograms performed under the supervision of a cardiologist.

Statistical analysis

The collected data were entered into Microsoft Excel (Microsoft, Redmond, WA) and analyzed using SPSS version 22 software (IBM Corp., Armonk, NY). Categorical data were presented as frequency and percentage. The significance of qualitative data was tested using the chi-square or Fisher's exact test. Continuous data were presented as the mean and standard deviation. A p-value < 0.05 was considered statistically significant. Receiver operating characteristic (ROC) curves were used to assess the validity of lipid profile values and ratios, with the area under the curves (AUC) calculated. Cut-off values, sensitivity, and specificity were also determined for lipid profile values and ratios.

## Results

The distribution of cases and controls is shown in Table 1.

**Table 1:** Distribution of cases and controls TGL: triglyceride lipase; CHO: cholesterol; mmHg: millimeters of mercury; mg/dL: milligrams per deciliter; N: number of patients.

Variables	Cases, N (%)	Controls, N (%)	p-value
Mean ± standard deviation, age (years)	52.18 ± 9.44	55.58 ± 8.94	0.067
Gender			
Male	13 (26%)	22 (44%)	0.059
Female	37 (74%)	28 (56%)	
Total	50 (100%)	50 (100%)	
Age group (years)			
31-40	6 (12%)	2 (4%)	0.440
41-50	20 (40%)	16 (32%)	
51-60	13 (26%)	16 (32%)	
61-70	10 (20%)	14 (28%)	
71-80	1 (2%)	2 (4%)	
Total	50 (100%)	50 (100%)	
Co-morbidities (hypertension, diabetes, and obesity)			
Present	35 (70%)	24 (48%)	0.025
Absent	15 (30%)	26 (52%)	
Total	50 (100%)	50 (100%)	
Blood pressure (in mmHg)			
Mean systolic blood pressure (SBP)	127.00	123.00	0.286
Standard deviation SBP	21.78	14.88	
Mean diastolic blood pressure (DBP)	78.60	79.20	0.790
Standard deviation DBP	11.61	10.85	
Blood glucose (mg/dL)			
Mean	169.12	165.80	0.786
Standard deviation	72.98	45.58	
Smoking	Cases (%)	Controls (%)	p-value
Present	26 (52%)	17 (34%)	0.069
Absent	24 (48%)	33 (66%)	
Total	50 (100%)	50 (100%)	
Cardiovascular examination (CVS)			
Pan systolic murmur (PSM)	4 (8%)	2 (4%)	0.652
Mid diastolic murmur (MDM)	2 (4%)	1 (2%)	
Ejection systolic murmur (ESM)	1 (2%)	1 (2%)	
Loud P2	1 (2%)	1 (2%)	
Loud S1	1 (2%)	0 (0%)	
Normal	41 (82%)	45 (90%)	
Total	50 (100%)	50 (100%)	
ECG findings (electrocardiogram)			
Ischemic heart disease (IHD)	45 (90%)	0 (0%)	<0.001
Left bundle branch block (LBBB)	3 (6%)	0 (0%)	
Left ventricular hypertrophy (LVH)	1 (2%)	0 (0%)	
Atrial fibrillation (AF)	1 (2%)	0 (0%)	
Normal	0 (0%)	50 (100%)	
Total	50 (100%)	50 (100%)	
Total bilirubin (mg/dL)			
Mean	0.64	0.72	0.105
Standard deviation	0.27	0.20	
Lipid profile (mg/dl)			
Mean total cholesterol (TC)	179.66	144.66	<0.001
Standard deviation TC	44.54	37.48	
Mean high-density lipoprotein (HDL)	36.84	52.88	<0.001
Standard deviation HDL	7.76	6.91	
Mean low-density lipoprotein (LDL)	119.42	76.72	<0.001
Standard deviation LDL	34.04	13.05	
Mean triglycerides	161.74	112.34	<0.001
Standard deviation TGL	48.97	23.81	
Lipid ratio			
Mean CHO/HDL	5.00	3.34	0.008
Standard deviation	1.44	1.07	
Mean LDL/HDL	3.30	1.48	<0.001
Standard deviation	0.96	0.35	
Mean HDL + bilirubin	101.26	124.90	<0.001
Standard deviation	28.28	21.92	
Mean LDL/(HDL + bilirubin)	1.21	0.61	<0.001
Standard deviation	0.49	0.15	
Mean TC/(HDL + bilirubin)	1.93	1.14	<0.001
Standard deviation	0.71	0.38	

There was no significant difference in age between the two groups (cases and controls), with a p-value of 0.067. The age distribution was comparable between the groups. The gender distribution was not significantly different between cases and controls (p-value = 0.059). The proportion of females was higher in the case group compared to the controls. The distribution of cases and controls across different age categories did not show a significant association with case or control status (p-value = 0.440). The age distribution was similar between the two groups. Lipid ratios (cholesterol (CHO)/HDL, LDL/HDL, HDL + bilirubin, LDL/(HDL + bilirubin), and TC/(HDL + bilirubin)) showed promising potential as predictors of CAD, outperforming traditional lipid profile parameters and total bilirubin levels. These ratios could serve as valuable diagnostic tools in identifying individuals at higher risk of CAD, especially in those with comorbidities such as hypertension, diabetes, and obesity. The presence of comorbidities was significantly higher in cases compared to controls (p-value = 0.025). This suggests that comorbidities may be associated with an increased risk of CAD. There was no significant difference in systolic blood pressure (SBP) and diastolic blood pressure (DBP) between cases and controls (p-values = 0.286 and 0.790, respectively). Blood glucose levels did not differ significantly between cases and controls (p-value = 0.786). Although the difference in the proportion of smokers between cases and controls was not statistically significant (p-value = 0.069), a higher percentage of cases had a history of smoking compared to controls.

The distribution of cases and controls according to cardiovascular (CVS) examination findings revealed that cardiac examination outcomes were comparable between the cases and controls. A p-value of 0.652 indicates that there was no statistically significant difference. The presence of ischemic heart disease (IHD) in the ECG was significantly higher in cases compared to controls (p-value < 0.001), indicating that IHD findings are strongly associated with CAD. Total bilirubin levels did not show a statistically significant difference between cases and controls (p-value = 0.105). Total bilirubin alone was not associated with CAD in this study. Lipid profile parameters (TC, HDL, LDL, and triglycerides) were significantly different between cases and controls (p-values < 0.001), indicating their association with CAD. Lipid ratios (CHO/HDL, LDL/HDL, HDL + bilirubin, LDL/(HDL + bilirubin), and TC/(HDL + bilirubin)) were significantly different between cases and controls (p-values < 0.001), suggesting that these ratios may serve as potential predictors of CAD (Table 1).

ROC curve analysis for lipid ratios demonstrated that the lipid ratios (CHO/HDL, LDL/HDL, LDL/(HDL + bilirubin), and TC/(HDL + bilirubin)) had high AUC values, indicating their good diagnostic performance in identifying CAD (Table 2).

**Table 2:** ROC curve analysis for lipid ratios CHO: cholesterol; HDL: high-density lipoprotein; LDL: low-density lipoprotein; TC: total cholesterol; AUC: area under the curve; ROC: receiver operating characteristic curve; CI: confidence interval.

Lipid ratio	AUC	p-value	95% CI (lower bound)	95% CI (upper bound)
CHO/HDL	0.913	<0.001	0.856	0.970
LDL/HDL	0.977	<0.001	0.955	1.000
LDL/(HDL + bilirubin)	0.903	<0.001	0.833	0.973
TC/(HDL + bilirubin)	0.807	<0.001	0.720	0.895

The assessment of lipid ratios' sensitivity and specificity, along with the derived cut-off values, underscores their potential as valuable diagnostic instruments for identifying individuals who are at a higher risk of developing CAD (Table 3).

**Table 3:** Sensitivity and specificity of lipid ratios CHO: cholesterol; HDL: high-density lipoprotein; LDL: low-density lipoprotein; TC: total cholesterol.

Lipid ratio	Cut-off	Sensitivity (%)	Specificity (%)
CHO/HDL	3.480	92	76
LDL/HDL	1.815	94	84
LDL/(HDL + bilirubin)	0.903	90	82
TC/(HDL + bilirubin)	1.275	86	64

ROC curve analysis for total bilirubin showed a non-significant AUC value (p-value = 0.119), indicating that total bilirubin alone may not be a reliable predictor of CAD (Table 4).

**Table 4:** ROC curve analysis for total bilirubin AUC: area under the curve; ROC: receiver operating characteristic curve; CI: confidence interval.

Total bilirubin	AUC	p-value	95% CI (lower bound)	95% CI (upper bound)
Total bilirubin	0.590	0.119	0.447	0.704

Total bilirubin demonstrated moderate sensitivity but low specificity in identifying CAD at the calculated cut-off value of 0.59 (Table 5).

**Table 5:** Sensitivity and specificity of total bilirubin

Total bilirubin	Cut-off	Sensitivity (%)	Specificity (%)
Total bilirubin	0.59	82	48

In summary, the study found that comorbidities, ECG findings, and lipid profile parameters were significantly associated with CAD. Among the lipid ratios, CHO/HDL, LDL/HDL, HDL + bilirubin, LDL/(HDL + bilirubin), and TC/(HDL + bilirubin) showed promising potential as predictors of CAD, outperforming total bilirubin alone. These findings may help in improving risk assessment and diagnosis of CAD.

## Discussion

In the present study, we investigated the association of various risk factors with CAD in a case-control setting. Our findings provide valuable insights into the potential risk factors contributing to CAD development. To contextualize our results, we compared them with those of previous valid studies, and the following discussion highlights the implications of our findings.

Our study observed a non-significant difference in mean age between cases and controls, and the majority of subjects in both groups were in the age group of 41-50 years. This finding is consistent with the study by Goode et al. [11], which also reported no significant age difference between cases and controls. Moreover, age-matching was performed in both studies, reducing the potential confounding effect of age on CAD risk.

Our findings demonstrate a higher prevalence of co-morbidities, such as diabetes and hypertension, in CAD cases compared to controls, consistent with previous studies [12,13]. These observations reinforce the well-established association between these co-morbid conditions and the development of CAD. Although the difference in systolic blood pressure between cases and controls was not statistically significant, the trend toward elevated systolic blood pressure aligns with the findings of Goode et al. [11].

The higher percentage of smokers among CAD cases in our study aligns with previous valid studies [14], indicating smoking is a significant risk factor for CAD development. However, it is important to note that the difference in smoking history between cases and controls in our study was not statistically significant, which could be attributed to the relatively small sample size.

Our study's findings regarding dyslipidemia, with higher total cholesterol, LDL, and triglycerides and lower HDL levels in CAD cases, are consistent with numerous previous studies [14-17]. This supports the well-established role of lipid abnormalities as major risk factors for CAD development.

Our study found an inverse correlation between serum total bilirubin levels and CAD risk, although the difference was not statistically significant [18,19]. However, this finding is in line with various previous studies [12-14] that reported a protective effect of bilirubin against CAD development. The antioxidant properties of bilirubin refer to its ability to neutralize harmful molecules known as free radicals within the body. Free radicals can damage cells and contribute to various health conditions, including atherosclerosis, a disease characterized by the buildup of fatty deposits in the arteries. When there is an excess of free radicals in the bloodstream, they can oxidize LDL, commonly referred to as "bad" cholesterol. This oxidized LDL is a key player in the development of atherosclerosis. It triggers inflammation and the formation of plaques on the artery walls, narrowing the blood vessels and restricting blood flow. Bilirubin, as an antioxidant, helps counteract this process by neutralizing free radicals and preventing the oxidation of LDL cholesterol. In doing so, it may reduce the inflammatory response and slow down the progression of atherosclerosis. The antioxidant properties of bilirubin have been suggested to play a role in its potential protective effect against atherosclerosis [15,20,21].

In our study, total bilirubin was added to lipid ratios like TC/(HDL + bilirubin) and LDL/(HDL + bilirubin) to make them better at diagnosing CAD. These results are in line with what Schwertner et al. [22] found, which suggests that adding bilirubin to lipid ratios improves their ability to predict the risk of CAD.

Overall, our study provides valuable insights into the association of various risk factors with CAD development in a case-control setting. The non-significant differences observed in some parameters may be attributed to the relatively small sample size and warrant further investigation with larger cohorts. Also, the results of total bilirubin and its effect on lipid ratios need to be looked at in more detail to find out if they could protect against CAD.

Limitations of the study include a smaller sample size, retrospective design, and a highly selected patient population. However, it is important to note that our study did not include age and gender matching between cases and controls, which could introduce potential biases. The observed associations between lipid ratios and CAD risk should be interpreted in consideration of these demographic differences.

## Conclusions

Our study provides valuable insights into the association of various risk factors with CAD. The findings corroborate the importance of traditional risk factors, such as dyslipidemia and smoking, as well as the potential protective role of bilirubin against CAD development. By comparing our results to those of other valid studies, we show how important it is to look at multiple risk factors when figuring out CAD risk and how adding bilirubin to lipid ratios could make them more accurate predictors. Nevertheless, more extensive research with larger, well-designed cohorts is necessary to validate these findings and further understand the complex interplay of risk factors in CAD pathogenesis.
